# Morphological and metabolic asymmetries of the thalamic subregions in temporal lobe epilepsy predict cognitive functions

**DOI:** 10.1038/s41598-023-49856-x

**Published:** 2023-12-18

**Authors:** Hsin Tung, Shih-Chuan Tsai, Pu-Rong Huang, Peiyuan F. Hsieh, Yi-Ching Lin, Syu-Jyun Peng

**Affiliations:** 1 Department of Post-Baccalaureate Medicine, College of Medicine, National Chung Hsing University, Taichung, Taiwan; 2 Center of Faculty Development, Taichung Veterans General Hospital, Taichung, Taiwan; 3 Neurological Institute, Taichung Veterans General Hospital, Taichung, Taiwan; 4 Institute of Clinical Medicine, National Yang Ming Chiao Tung University, Taipei, Taiwan; 5 Department of Nuclear Medicine, Taichung Veterans General Hospital, Taichung, Taiwan; 6 Department of Medical Imaging and Radiological Technology, Institute of Radiological Science, Central Taiwan University of Science and Technology, Taichung, Taiwan; 7 Professional Master Program in Artificial Intelligence in Medicine, College of Medicine, Taipei Medical University, No.250, Wuxing St., Xinyi Dist., Taipei City, 110 Taiwan; 8 Clinical Big Data Research Center, Taipei Medical University Hospital, Taipei Medical University, Taipei, Taiwan

**Keywords:** Neuroscience, Neurology

## Abstract

Both morphological and metabolic imaging were used to determine how asymmetrical changes of thalamic subregions are involved in cognition in temporal lobe epilepsy (TLE). We retrospectively recruited 24 left-TLE and 15 right-TLE patients. Six thalamic subnuclei were segmented by magnetic resonance imaging, and then co-registered onto Positron emission tomography images. We calculated the asymmetrical indexes of the volumes and normalized standard uptake value ratio (SUVR) of the entire and individual thalamic subnuclei. The SUVR of ipsilateral subnuclei were extensively and prominently decreased compared with the volume loss. The posterior and medial subnuclei had persistently lower SUVR in both TLE cases. Processing speed is the cognitive function most related to the metabolic asymmetry. It negatively correlated with the metabolic asymmetrical indexes of subregions in left-TLE, while positively correlated with the subnuclei volume asymmetrical indexes in right-TLE. Epilepsy duration negatively correlated with the volume asymmetry of most thalamic subregions in left-TLE and the SUVR asymmetry of ventral and intralaminar subnuclei in right-TLE. Preserved metabolic activity of contralateral thalamic subregions is the key to maintain the processing speed in both TLEs. R-TLE had relatively preserved volume of the ipsilateral thalamic volume, while L-TLE had relatively decline of volume and metabolism in posterior subnucleus.

Although the thalamus is a subcortical structure, it contains several cytoarchitectonically distinct neurons, having reciprocal projections toward widespread cortical regions^1^. It regulates or transmits neuronal information between or within brain regions, determining sensory, motor, behavioral, and cognitive processes^2^ and is thought as a hub for controlling functional connectivity for the computation of executive functions^3^.

Epilepsy activities modify the specific brain connectivity and then reorganize the connectivity network in both ictal and interictal periods of temporal lobe epilepsy (TLE)^4^. Thalamo-hippocampal synchronization is augmented in the early phase of epilepsy, which is associated with the period of irregular spiking transition to the bursting phase^5^. Disrupted thalamocortical connectivity is not only involved in spike generation^6^, but also related to memory impairment in patients with TLE^7,8^. Interictal thalamocortical dysfunction is observed in drug-naïve TLE patients via the high-frequency oscillation of somatosensory evoked potentials^9^. The altered characteristics of the thalamus in TLE are also demonstrated in structural, functional, and metabolic imaging modalities respectively, like volume loss^10,11^, altered diffusion properties^12^, and hypometabolism^13^ in the ipsilateral thalamus.

The thalamus is divided into several subregions based on its projections and the location. Each subregion has different roles in seizure activity. The anterior nucleus mainly connects the medial temporal structure^14^, and modulates the seizure activity from the large cortical area. The midline thalamic nuclei are involved in seizure initiation and neuronal recruitment during the ictal period^5,15^. Differential nucleus activation occurs in different phases of cognitive processing, including learning and retrieval^16^. These subnuclei also exhibit differential vulnerability to seizure activity. The pulvinar and anterior nuclei show impaired BOLD signals in TLE, because they are densely connected to the hippocampus^17^. Nevertheless, there were no comprehensive studies on how epilepsy activity reconfigures the networks of each thalamic subregion and how it alters cognition.

Positron emission tomography (PET) imaging uses the glucose analog^18^F-fluorodeoxyglucose (FDG) to measure the status of glucose metabolism in cells^18^. Local energy consumption and efficacy of the synaptic transmission are quantified by using standard uptake values (SUVs), which are the semi-quantitative units of PET intensity. SUVs are equal to the radioactivity concentration of the focal region divided by the injected dose, and then further divided by the body weight, which are independent of neurovascular coupling^19^. Brain regions presenting the similar SUVs indicated that they have functional coupling and metabolic covariance, which match the underlying structural connectivity^19^. Focal metabolic deficits on the PET images of patients with epilepsy are generally used for assisting identification of the epileptogenic zones for presurgical evaluation, in addition to video-electroencephalography (vEEG) and brain magnetic resonance imaging (MRI). Moreover, the focal, asymmetrical hypometabolism of cortical regions maps the impairments of specific functions and cognitions^20^.

Cognitive impairment is a common comorbidity in patients with epilepsy^21^. It is related to both the seizure itself and other modifying factors, such as the duration and the type of the seizure, the epileptogenic focus, the control of the seizure, and the use of antiseizure medications (ASMs)^22^. A cognitive process is accomplished via the functional integration and segregation of multiple brain regions^23–25^. Therefore, any alterations in their connectivity among brain regions would perturb the neuropsychological performance. TLE causes extensive disruption of the cortical–subcortical networks, which is associated with multiple cognitive domain impairments^26,27^. Impaired intelligence quotient (IQ) scores in patients with TLE are associated with hypometabolism in regions involving the default mode network^28^, which suggests that the extratemporal connectivity is related to cognitive function. However, metabolic alterations in subcortical structures still have an undisclosed implication in cognitive functions.

Currently, the roles of the thalamus in TLE and the cognitive networks have been gradually clarified. However, how individual thalamic subregions are altered by epilepsy has not been well identified, neither their differential contribution to cognitive functions has been elucidated. Therefore, we used both morphological and metabolic imaging data to examine the relationship between the degree of the asymmetry of thalamic subregions and the cognitive performance in patients with TLE.

## Materials and methods

### Participants

We conducted this retrospective study, by enrolling patients with TLE, who underwent evaluations for epilepsy surgery from August, 2019 to September, 2020 at Taichung Veterans General Hospital (TCVGH). All methods were performed in accordance with the relevant guidelines and regulations. Their epilepsy histories were obtained, and they at least completed the following comprehensive phase I studies: vEEG, high-resolution brain MRI, PET imaging, and neuropsychological evaluations. In concern of altered thalamic connectivity in focal to bilateral tonic–clonic seizures^29^, we selected patients who received these exams at the time when no secondarily generalized tonic–clonic seizures occurred for at least 6 months. Patients with brain tumors, severe psychiatric disorders, a history of head trauma or stroke, and temporal-plus epilepsy were excluded. This study was approved by the Institutional Review Board of TCVGH (CE18306B). The informed consent was obtained from all subjects and/or their legal guardian(s).

### Neuropsychological evaluations

Neuropsychiatric tests were conducted within one month before or after the acquisition of brain imaging, and performed by an experienced psychologist. The four main domains of IQ were evaluated using the Wechsler Adult Intelligence Scale III (WAIS version III). The following five scores were extracted for analysis: Full-scale IQ (FIQ), Verbal IQ (VIQ), Performance IQ (PIQ), Working Memory Index (WMI), and Processing Speed Index (PSI). WMI measures the subjects how to attend the information provided verbally, and then manipulate and formulate a response. PSI assesses the subjects how to perform the tasks involving attention and visual-motor coordination correctly and efficiently, by using graphomotor skills and visual stimuli.

### Imaging acquisition

PET images were acquired using Gemini TF PET/CT (Philips Medical Systems)^28^. ^18^F-FDG was administered intravenously at a dose of 5 MBq/kg after at least 6-h of fasting. After 30 min, brain images were taken in the static acquisition mode for 15 min. Ordered subset expectation maximization was used to iteratively reconstruct PET data onto the brain CT images. Subsequently, the 2 × 2 × 2 mm^3^ voxel size images were generated. MRI scans were obtained using a 1.5-T scanner (Aera, Siemens, Erlangen, Germany), equipped with a 20-channel phase-array head coil. The 3D-magnetization-prepared rapid acquisition with gradient-echo (MPRAGE) images were acquired using the following parameters: repetition time = 2800 ms, inversion time = 930 ms, echo time = 5.13 ms, spatial resolution = 0.8 × 0.8 × 1.0 mm^3^, and flip angle = 8°.

### Image processing and thalamus segmentation

**Figure 1:**
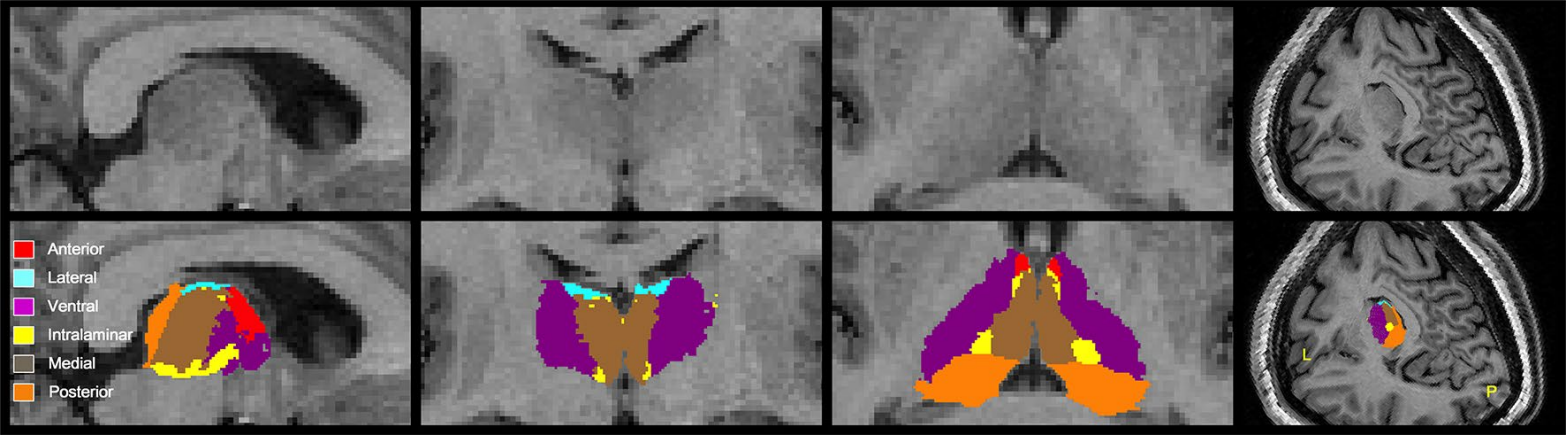
Localization of the six subregions of the thalamus: anterior, lateral, ventral, intralaminar, medial, and posterior. They are sagittal, coronal, axial, and 3D volume rendering views of the thalamic subnuclei from the left to right figures.

The^18^F-FDG PET images were co-registered with the MPRAGE images, and then segmented into the six subfields (Fig. 1). Their volumes and the SUVs were calculated individually. The detail methods were described in the “Supplementary materials”.

We assumed that thalamic volumes and metabolic activity were bilaterally equal in subjects without epilepsy. Therefore, the asymmetrical indices of each imaging data from each subregion were calculated by 2 × [(L − R)/(L + R)], which represented the degree of differences between both sides in volume and in SUVRs. We supposed suchasymmetric vulnerability was affected by epilepsy activity. The processing flow chart is depicted in Supplementary Fig. 1.

### Statistical analysis

The volume and normalized SUVs (SUV ratio, SUVRs) of each thalamic subfield on the ipsilateral and the contralateral sides were individually compared using the two-sided Wilcoxon signed rank test. Then, the false discovery rate was applied for correction, after initially identifying the more significantly different subregions. The asymmetrical index was calculated for volumes and SUVRs of each subregion. The relationship between each IQ score and each asymmetrical index of the individual subregion in both imaging modalities was determined using Spearman partial correlations. Age was counted as the covariate. Differences were considered significant when *p* values were less than 0.05. All statistical analyses were two-tailed, and calculated using MATLAB R2021a (MathWorks Inc., Natick, Massachusetts).

## Results

We identified 24 patients with left-TLE (L-TLE) and 15 patients with right-TLE (R-TLE). The demographic characteristics of both groups showed no statistically significant differences (Table 1), including age, gender, seizure duration, seizure onset age, educational years, the number of ASMs, and individual IQ scores. The ratio of ever secondary generalization, history of febrile convulsion, the configurations of hippocampal morphologies (sclerosis, normal, or enlargement) were also similar in both groups. We excluded one of the L-TLE patients for calculating the correlation between the asymmetrical indices and the four main IQ scores because he did not complete the comprehensive neuropsychiatric exam.

**Table 1:** Demographic characteristics of left-TLE and right-TLE cases.

	L-TLE (n = 24) Median (Quartiles)	R-TLE (n = 15) Median (Quartiles)	*p*^&^
Study age	35.3 (28.8–47.7)	40.0 (27.3–54.7)	0.539
Seizure onset age	23.0 (15.0–29.8)	20.0 (13.0–33.0)	0.921
Gender (Male:Female)	10:14	7:8	0.760
Seizure duration (years)	13.0 (6.5–20.8)	11.0 (6.0–42.0)	0.658
Educational years	16 (12–16)	13 (9–16)	0.172
FIQ	78 (73–94)	82 (66–90)	0.616
VIQ	84 (70–93)	80 (70–99)	0.860
PIQ	84 (72–96)	78 (74–106)	0.555
WMI	86 (75–97)	82 (68–99)	0.555
PSI	84 (77–94)	84 (68–94)	0.680
Numbers of ASMs	3 (2–3)	3 (2–4)	0.966
Using VPA, ZNS, or PHT	11 (45.8%)	8 (53.3%)	0.6480.648
Ever secondary generalization	13 (54.2%)	8 (53.3%)	0.9590.959
History of febrile convulsion	4 (16.7%)	1 (6.7%)	0.6770.677
Hippocampal sclerosis	11 (45.8%)	7 (47.7%)	0.9590.959
Hippocampal enlargement	2 (8.3%)	1 (6.7%)	0.6420.642
Seizure frequency (per month)^#^	1.5 (1.0–5.0)	2.5 (1.0–4.0)	0.853

*FSIQ* Full-Scale IQ; *VIQ* Verbal IQ; *PIQ* Performance IQ; *WMI* Working Memory Index; *PSI* Processing Speed Index; *ASM* anti-seizure medication; *VPA* valproic acid; *ZNS* Zonisamide; *PHT* Phenytoin.

^&^Mann–Whitney U test & Chi-squared.

^#^It was defined by the average of monthly seizure number in the past 6 months of the date of neuropsychiatric test.

### Asymmetrical volume and metabolic changes in the thalamic subnuclei in both TLE cases

Morphologically, the ipsilateral entire thalamus showed a significantly smaller volume compared with the volume of the contralateral thalamus in both TLE cases. Comparison between the volumes of thalamic subregions on both sides in patients with L-TLE (Table 2) showed that only the anterior (*p* = 0.042) and posterior (*p* = 0.015) nuclei were significantly smaller on the ipsilateral side. Metabolically, the SUVR were significantly decreased in the ipsilateral intralaminar (*p* = 0.001), medial (*p* = 0.007), and posterior (*p* = 0.001) subnuclei.

**Table 2:** Comparison of volumes and SUVR of six thalamic subnuclei of the ipsilateral and contralateral sides in patients with left-TLE.

	Volume (mm^3^) (mean ± SD)				SUVR (mean ± SD)			
	Ipsilateral (left)	Contralateral (right)	*p*^$^	Adjusted* p*^#^	Ipsilateral (left)	Contralateral (right)	*p*^$^	Adjusted *p*^#^
Anterior	139.698 ± 31.667	149.224 ± 26.251	0.014*	0.042*	0.497 ± 0.181	0.505 ± 0.179	0.775	0.841
Lateral	157.386 ± 40.859	159.863 ± 33.772	0.568	0.728	0.686 ± 0.244	0.672 ± 0.214	0.841	0.841
Ventral	2952.537 ± 500.024	2986.522 ± 420.576	0.607	0.728	0.292 ± 0.112	0.305 ± 0.112	0.424	0.636
Intralaminar	443.103 ± 81.987	447.328 ± 65.982	0.954	0.954	0.553 ± 0.116	0.598 ± 0.124	< 0.001**	0.001**
Medial	991.613 ± 128.559	1014.956 ± 102.898	0.110	0.219	0.964 ± 0.205	0.991 ± 0.200	0.003**	0.007**
Posterior	2263.163 ± 353.714	2420.401 ± 332.335	0.002**	0.015*	0.661 ± 0.157	0.708 ± 0.137	<0.001**	0.001**
Whole thalamus	6947.500 ± 1056.428	7178.293 ± 875.606	0.040*		0.537 ± 0.136	0.567 ± 0.131	0.001**	

*SD* standard deviation, *SUVR* standard uptake value ratio.

*p < 0.05, **p < 0.01

^**$**^Two-sided Wilcoxon signed rank test.

^#^Corrected by using multiple comparison by false discovery rate (FDR).

In patients with R-TLE, there was no significant volume asymmetry in the thalamic subregions (Table 3). However, PET imaging demonstrated significantly lower SUVs mainly in the anterior nucleus (*p* = 0.016), followed by the posterior (*p* = 0.031) and medial (*p* = 0.043) subnuclei. In both TLE cases, the entire thalamus showed a significant decrease in both total volumes and SUVR ipsilaterally.

**Table 3:** Comparison of volumes and SUVRs of six thalamic subnuclei of the ipsilateral and contralateral sides in patients with right-TLE.

	Volume (mm^3^) (mean ± SD)				SUVR (mean ± SD)			
	Ipsilateral (right)	Contralateral (left)	*p*^$^	Adjusted *p*^#^	Ipsilateral (right)	Contralateral (left)	*p*^$^	Adjusted *p*^#^
Anterior	131.152 ± 17.566	134.547 ± 15.935	0.208	0.344	0.469 ± 0.092	0.511 ± 0.088	0.003**	0.016*
Lateral	143.154 ± 29.421	148.295 ± 20.209	0.229	0.344	0.598 ± 0.135	0.621 ± 0.119	0.303	0.363
Ventral	2666.269 ± 326.651	2753.129 ± 342.273	0.015	0.090	0.313 ± 0.065	0.334 ± 0.080	0.151	0.227
Intralaminar	400.736 ± 52.426	403.994 ± 54.671	0.524	0.629	0.618 ± 0.084	0.613 ± 0.081	0.639	0.639
Medial	954.315 ± 113.193	975.617 ± 94.572	0.169	0.344	0.928 ± 0.104	0.967 ± 0.085	0.022*	0.043*
Posterior	2132.233 ± 294.784	2136.551 ± 273.283	0.934	0.934	0.625 ± 0.079	0.669 ± 0.092	0.010*	0.031*
Whole thalamus	6427.859 ± 774.444	6552.132 ± 745.348	0.010*		0.536 ± 0.069	0.564 ± 0.076	0.035*	

*SD* standard deviation, *SUVR* standard uptake value ratio.

*p < 0.05, **p < 0.01

^$^Two-sided Wilcoxon signed rank test.

^#^Corrected by using multiple comparison by false discovery rate (FDR).

### Volume alterations related to epilepsy duration and metabolism changes related to PSI in L-TLE

Regarding the four cognitive domains, only VIQ correlated with structural images in patients with L-TLE. Preserved left anterior subnucleus volumes demonstrated better VIQ (*p* = 0.001) (Table 4). In the metabolic imaging, only the PSI domain negatively correlated with the asymmetrical index of SUVR of most subregions (Table 5), including lateral (*p* = 0.002), intralaminar (*p* = 0.002), and medial (*p* = 0.019). Thus, patients with L-TLE, who had compensated increase of right thalamic metabolism, possessed better PSI.

**Table 4:** Relationship between the asymmetry index of thalamic subnuclei volume and neurocognitive scores in patients with (a) left-TLE and (b) right-TLE; correlation coefficient (*p* value).

(a) Asymmetry index	Anterior	Lateral	Ventral	Intralaminar	Medial	Posterior	Whole thalamus
FSIQ	0.408 (0.059)	0.277 (0.211)	− 0.043 (0.849)	− 0.015 (0.948)	− 0.054 (0.812)	− 0.055 (0.808)	− 0.040 (0.859)
VIQ	0.636 (0.001**)	0.357 (0.103)	0.285 (0.199)	0.255 (0.253)	0.168 (0.455)	0.067 (0.766)	0.214 (0.339)
PIQ	0.305 (0.168)	0.196 (0.381)	− 0.195 (0.385)	− 0.095 (0.675)	− 0.099 (0.662)	− 0.099 (0.660)	− 0.136 (0.546)
WMI	0.412 (0.057)	0.242 (0.278)	0.043 (0.850)	0.074 (0.745)	0.143 (0.526)	0.078 (0.729)	0.086 (0.704)
PSI	− 0.015 (0.948)	0.216 (0.335)	− 0.053 (0.815)	− 0.085 (0.705)	− 0.442 (0.040)	− 0.234 (0.295)	− 0.159 (0.480)
Onset age	0.274 (0.194)	0.322 (0.125)	0.253 (0.233)	0.318 (0.129)	0.104 (0.627)	0.269 (0.204)	0.321 (0.126)
Disease duration	− 0.392 (0.058)	− 0.387 (0.062)	− 0.458 (0.024*)	− 0.467 (0.021*)	− 0.488 (0.016*)	− 0.432 (0.035*)	− 0.525 (0.008**)

(b) Asymmetry index	Anterior	Lateral	Ventral	Intralaminar	Medial	Posterior	Whole thalamus
FSIQ	0.413 (0.142)	0.226 (0.437)	0.267 (0.356)	0.072 (0.808)	0.278 (0.336)	0.450 (0.106)	0.557 (0.039*)
VIQ	0.455 (0.102)	0.359 (0.208)	0.015 (0.960)	0.026 (0.929)	0.178 (0.542)	0.358 (0.209)	0.326 (0.255)
PIQ	0.447 (0.109)	0.328 (0.252)	0.197 (0.499)	0.156 (0.594)	0.275 (0.342)	0.476 (0.086)	0.538 (0.047*)
WMI	0.251 (0.387)	0.162 (0.581)	0.291 (0.313)	0.148 (0.614)	0.200 (0.493)	0.574 (0.032*)	0.615 (0.019*)
PSI	0.695 (0.006**)	0.431 (0.124)	0.109 (0.711)	− 0.037 (0.900)	0.146 (0.619)	− 0.046 (0.877)	0.210 (0.470)
Onset age	0.289 (0.295)	0.311 (0.259)	− 0.186 (0.507)	− 0.279 (0.314)	0.136 (0.630)	0.236 (0.397)	0.314 (0.254)
Disease duration	− 0.005 (0.985)	− 0.328 (0.233)	0.450 (0.093)	− 0.088 (0.756)	− 0.306 (0.267)	− 0.341(0.214)	− 0.203 (0.469)

*FSIQ* Full-Scale IQ; *VIQ* Verbal IQ; *PIQ* Performance IQ; *WMI* Working Memory Index; *PSI* Processing Speed Index.

*p < 0.05; **p < 0.01.

Spearman partial correlations, age as covariate.

Asymmetrical index = 2x [(L − R)/(L + R)].

**Table 5:** Relationship between the asymmetry index of thalamic subnuclei SUVR and neurocognitive scores in patients with (a) left-TLE and (b) right-TLE; correlation coefficient (*p* value).

(a) Asymmetry index	Anterior	Lateral	Ventral	Intralaminar	Medial	Posterior	Whole thalamus
FSIQ	0.037 (0.869)	− 0.346 (0.115)	0.058 (0.798)	− 0.176 (0.432)	− 0.277 (0.211)	− 0.259 (0.244)	− 0.173 (0.442)
VIQ	0.228 (0.308)	− 0.162 (0.472)	− 0.094 (0.678)	0.074 (0.743)	0.141 (0.533)	0.142 (0.527)	0.043 (0.850)
PIQ	− 0.141 (0.532)	− 0.378 (0.083)	0.105 (0.641)	− 0.234 (0.295)	− 0.409 (0.059)	− 0.305 (0.167)	− 0.208 (0.352)
WMI	0.250 (0.262)	− 0.172 (0.445)	0.181 (0.421)	0.063 (0.779)	− 0.089 (0.694)	− 0.320 (0.147)	0.025 (0.910)
PSI	− 0.350 (0.111)	− 0.627 (0.002**)	− 0.288 (0.193)	− 0.618 (0.002**)	− 0.494 (0.019*)	− 0.409 (0.059)	− 0.648 (0.001**)
Onset age	0.057 (0.790)	− 0.174 (0.415)	− 0.066 (0.760)	− 0.021 (0.923)	− 0.224 (0.292)	0.027 (0.902)	0.010 (0.965)
Disease duration	− 0.315 (0.134)	0.233 (0.273)	− 0.053 (0.805)	0.231 (0.279)	− 0.008 (0.969)	− 0.205 (0.336)	− 0.139 (0.516)

(b) Asymmetry index	Anterior	Lateral	Ventral	Intralaminar	Medial	Posterior	Whole thalamus
FSIQ	0.137 (0.641)	0.060 (0.839)	0.104 (0.722)	0.179 (0.540)	0.376 (0.186)	− 0.032 (0.915)	0.216 (0.457)
VIQ	− 0.043 (0.883)	− 0.145 (0.622)	0.236 (0.416)	0.330 (0.249)	0.363 (0.203)	0.114 (0.698)	0.356 (0.212)
PIQ	0.001 (0.997)	− 0.122 (0.677)	0.059 (0.841)	0.175 (0.549)	0.273 (0.346)	0.021 (0.944)	0.188 (0.519)
WMI	− 0.048 (0.871)	− 0.027 (0.928)	− 0.167 (0.569)	− 0.058 (0.845)	0.105 (0.722)	− 0.165(0.573)	− 0.087 (0.768)
PSI	0.164 (0.576)	0.199 (0.496)	0.431 (0.124)	0.515 (0.060)	0.652 (0.012*)	0.212 (0.467)	0.598 (0.024*)
Onset age	− 0.032 (0.913)	0.018 (0.954)	0.411 (0.130)	0.321 (0.242)	0.371 (0.173)	0.057 (0.842)	0.314 (0.254)
Disease duration	− 0.215 (0.441)	− 0.072 (0.800)	− 0.668 (0.006**)	− 0.590 (0.021*)	− 0.297 (0.282)	− 0.324 (0.238)	− 0.663 (0.007**)

*FSIQ* Full-Scale IQ; *VIQ* Verbal IQ; *PIQ* Performance IQ; *WMI* Working Memory Index; *PSI* Processing Speed Index.

Significances values in Bold.

*p < 0.05; **p < 0.01.

Spearman partial correlations, age as covariate.

Asymmetrical index = 2x [(L − R)/(L + R)].

The duration of epilepsy was negatively related to asymmetrical indexes of thalamic volume, rather than metabolic alterations in patients with L-TLE. A longer epilepsy duration resulted in relatively smaller volumes of left ventral (*p* = 0.024), intralaminar (*p* = 0.021), medial (*p* = 0.016), and posterior (*p* = 0.035) subnuclei, as well as the entire left thalamus (*p* = 0.008).

### Volume changes related to some cognitive functions; whereas metabolism alterations related to epilepsy duration and PSI in R-TLE

In patients with R-TLE, the relatively larger left anterior subnuclei volume showed a better processing speed (*p* = 0.006), whereas the left posterior subnucleus exerted less influence (*p* = 0.032) on working memory (Table 4). Structurally, the relatively larger left total thalamic volume correlated with better FIQ (*p* = 0.039), PIQ (*p* = 0.047), and WMI (*p* = 0.019) performance. The metabolic images correlated with only the processing speed. Patients with higher SUVR of the left medial subnucleus (*p* = 0.012) and the entire left thalamus (*p* = 0.024) showed relatively better PSI (Table 5).

Epilepsy duration in patients with R-TLE was only associated with changes in thalamic metabolic asymmetry rather than volumes. SUVR was relatively decreased in the entire left thalamus (*p* = 0.007), as well as left ventral (*p* = 0.006) and intralaminar (*p* = 0.021) subnuclei in patients with longer epilepsy duration. However, the thalamic volumes remained unaffected.

## Discussion

In this study, we identified how the thalamic subnuclei reconfigurated in both structural and metabolic aspects in patients with R-TLE and L-TLE. We also identified that individual thalamic subnuclei not only participated in different cognitive functions, but also exhibited a differential vulnerability to epilepsy insults. These findings also revealed that mechanisms by which thalamic subregions reorganize in TLE would result in the different patterns of cognitive alterations.

Morphologically, our study revealed that both cases of TLE showed a relatively smaller entire and some subregions of thalamic volume on the ipsilateral side than those of the contralateral side. This phenomenon is expected because previous studies had demonstrated a decrease in bilateral neocortical and subcortical volumes in TLE^30,31^. The ipsilateral structures appeared to be more vulnerable^32^, which were believed to be a part of an epileptic network that functionally connected to the hippocampus. Deep GMs, like the thalamus and the pallidum, might be bilaterally affected^33^, which is related to their bilateral projections. Our study demonstrated the asymmetrical nature of the changes of the thalamus volume in TLE.

Among the six thalamic subnuclei, the ipsilateral posterior part in L-TLE presented significant atrophy. The posterior thalamic portion primarily consists of the pulvinar, which has dense structural connections with the hippocampus^34,35^. The medial pulvinar participates in ictal spreading during hippocampus-originating seizures^35^. Therefore, the significantly smaller ipsilateral posterior thalamus suggested its structural disruption of the thalamo-hippocampal pathway. This phenomenon echoed the functional MRI finding that the anterior and pulvinar subnuclei were most susceptible in TLE, presenting the increased band power of the specific frequency, especially on the ipsilateral side^17^. However, patients with R-TLE did not show obviously asymmetric volume changes of thalamic subregions rather than the entire volume. This indicated that thalamo-hippocampal connections in R-TLE were structurally stable, with relatively symmetrical volume of all thalamic subregions.

Metabolically, the ipsilateral medial and posterior thalamic subregions exhibited more significant hypometabolism in both our TLE cases. The mediodorsal nucleus is an important component of the medial thalamic subregion, connecting to the prefrontal and temporal cortexes in the diffusion imaging study^1^. Previous electrophysiological studies have demonstrated that the mediodorsal nucleus is involved in the seizure onset of the medial temporal lobe^8^, participating in the spread of seizures^36^. Therefore, seizure activity results in prominently lower metabolism in the ipsilateral medio-posterior thalamic subregion in our both L-TLE and R-TLE, which might be related to their dense connections to temporal lobes. This phenomenon could be used as a biomarker to assist in lateralizing the epileptogenic hippocampus. Furthermore, there are still some distinguishing characters of metabolic asymmetry. Patients with L-TLE showed an additional SUVR asymmetry in intralaminar subregion, whereas R-TLE had additionally relatively decreased SUVR in the ipsilateral anterior nucleus. This indicates that differential thalamic neurons are injured in cases of R-TLE and L-TLE, resulting in different metabolic networks.

In addition to memory impairment, processing speed is the cognitive domain that most prominently affected by epilepsy, even in drug-naïve patients^37,38^. Processing speed in TLE is related to altered volume integrity of white matter^39^, decreased cortical gyration, and decreased functional connectivity^40^. Among the four main cognitive domains, asymmetrical metabolism was mostly related to processing speed in our study. Slowed processing speed might further exert an impact on numerous cognitive functions in patients with TLE^38^. This phenomenon echoed our study finding, wherein processing speed is the most sensitive indicator among the four cognitive domains, correlated with the asymmetrical metabolic changes of thalamic subregions. PSI of both patients with L-TLE and R-TLE depended on the preserved metabolic activity of contralateral thalamic subregions, especially medial subregions. The medial thalamic network has been reported to be associated with familiarity and retrieval aspects of memory^41^, whose function is vulnerable to epilepsy activity.

Morphologically, only the anterior subnucleus was related to cognitive function in both cases of TLE in our study. The relatively preserved or larger left anterior nucleic volume suggested reserved competence, involving VIQ in L-TLE and processing speed in R-TLE. The anterior thalamic nucleus is suggested as a part of the salience network^42^, related to attention, memory processing, recollection and encoding in healthy subjects^43^. It is also the primary thalamic component connected to the hippocampus by the fornix, and its volume has a preferential vulnerability to age. The resting-state fMRI study also revealed that TLE patients demonstrated a disrupted thalamocortical network usually involving the anterior thalamus, which contributed to memory impairments^44^. Moreover, older healthy adults who have smaller anterior thalamic volumes was associated with impaired working memory and processing speed^45^. Therefore, it was expected that the decline in cognitive function in TLE might be reflected by the anterior thalamic volume.

Epilepsy duration had been reported to alter the network of TLE, reflecting not only on the hippocampus but also on the thalamus^46^. A longer duration of epilepsy has been reported to fray the thalamus in both morphological and metabolic aspects^10,47^. Our study further explored the differential vulnerability of the thalamic subregions. The thalamic volume of L-TLE presented prominent and diffuse atrophy of the ipsilateral thalamus in those had a longer epilepsy history, but the thalamic volume was relatively preserved in R-TLE. In contrast, only the thalamic metabolism was affected in R-TLE, whereas the metabolic activity was maintained in L-TLE, irrespective of the duration of epilepsy. This phenomenon revealed that the pattern of disrupted thalamic connectivity is different in patients with R-TLE and L-TLE.

Loss of brain volume on brain MRI suggested neuronal loss or decreased amount in glial cells, that suffered from irreversible cytotoxic insults^48^. However, the metabolic lesion on brain PET imaging shows a focal decrease in glucose uptake, indicating neuronal dysfunction. Epilepsy activity did not reorganize the neuronal volume and their glucose metabolism in the parallel trend. Metabolic activity can occur before the appearance of clinical symptoms^49^. This phenomenon was also observed in our study. Metabolic asymmetry was more prominent than volume asymmetry in both TLE cases. Both brain volume and metabolic declines were believed to be associated with many types of neurodegenerative disorders, and were also related to cognitive impairment. Brain areas with synchronously morphological or metabolic changes might comprise the neural network, which of them were present as different configurations. Partially reversible hypometabolism has been observed in patients with epilepsy after surgery^50^. However, whether postoperative recovery of cortical volume occurs after temporal lobectomy remains controversial^51,52^.

Our study has some limitations. First, there were no normal controls to directly compare the absolute values of volume and metabolic changes. Only relatively asymmetrical alterations were identified by comparing the corresponding subregions of the thalamus on both sides. It was difficult to clearly differentiate whether metabolic asymmetry is related to the contralateral compensatory mechanism or the ipsilateral hypofunction. Second, it was possible that ASMs also affected the cognitive function^53^. Some ASMs which bear the effects of blocking T-type calcium channels might suppress neuronal hypersynchrony in thalamus, which might affect the PET signals^54^. These impacts could not be totally concealed or be further grouped due to small sample size. These influences were similar in both TLE groups due to a similar number of ASMs used by the patients.

Additionally, the etiology of hippocampal sclerosis, normal, or enlarged hippocampus related TLE might have different network. However, due to small sample size, we only took them as one group for analysis. Further larger studies with matched controls, as well as more comprehensive neuropsychological functions are required in the future.

## Conclusion

Patients with L-TLE and those with R-TLE exhibited a differential morphological and metabolic vulnerability in their thalamic subregions. Metabolic asymmetry was more prominent than the volume differences, and the posterior and medial subnuclei presented persistently hypometabolism in both TLE groups. Thalamic subnuclei participated in cognitive functions, of which processing speed presented most relevant to asymmetrical metabolic changes of thalamic subregions in TLE. Preserved metabolic activity of contralateral thalamic subregions is the key to maintain the processing speed in both TLEs. R-TLE had relatively preserved volume of the ipsilateral thalamic volume, while L-TLE had relatively decline of volume and metabolism in posterior subnucleus. Longer epilepsy duration was associated with a prominent atrophy of most ipsilateral thalamic volume in L-TLE, but with a relative left thalamic hypometabolism in R-TLE.

### Supplementary Information

Supplementary Figure 1.

Supplementary Information 2.

## Data Availability

The datasets generated and/or analyzed during the current study are not publicly available due but are available from the corresponding author on reasonable request, because during data evaluation process all data has been anonymized.

## References

[1] Behrens TE, Johansen-Berg H, Woolrich MW (2003). Non-invasive mapping of connections between human thalamus and cortex using diffusion imaging. Nat. Neurosci..

[2] Nakajima M (2017). Halassa MM thalamic control of functional cortical connectivity. Curr. Opin. Neurobiol..

[3] Vien C, Bore A, Boutin A (2019). Thalamo-cortical white matter underlies motor memory consolidation via modulation of sleep spindles in young and older adults. Neuroscience.

[4] Moshe SL, Perucca E, Ryvlin P (2015). Epilepsy: New advances. Lancet.

[5] Aracri P, de Curtis M, Forcaia G (2018). Enhanced thalamo-hippocampal synchronization during focal limbic seizures. Epilepsia.

[6] Chiosa V, Groppa SA, Ciolac D (2017). Breakdown of thalamo-cortical connectivity precedes spike generation in focal epilepsies. Brain Connect..

[7] Voets NL, Menke RA, Jbabdi S (2015). Thalamo-cortical disruption contributes to short-term memory deficits in patients with medial temporal lobe damage. Cereb Cortex.

[8] Guye M, Regis J, Tamura M (2006). The role of corticothalamic coupling in human temporal lobe epilepsy. Brain.

[9] Assenza G, Lanzone J, Insola A (2020). Thalamo-cortical network dysfunction in temporal lobe epilepsy. Clin. Neurophysiol..

[10] Wu D, Chang F, Peng D (2020). The morphological characteristics of hippocampus and thalamus in mesial temporal lobe epilepsy. BMC Neurol..

[11] Tung H, Pan SY, Lan TH (2021). Characterization of hippocampal-thalamic-cortical morphometric reorganization in temporal lobe epilepsy. Front. Neurol..

[12] Gong G, Concha L, Beaulieu C (2008). Thalamic diffusion and volumetry in temporal lobe epilepsy with and without mesial temporal sclerosis. Epilepsy Res..

[13] Chassoux F, Artiges E, Semah F (2016). Determinants of brain metabolism changes in mesial temporal lobe epilepsy. Epilepsia.

[14] Sherdil A, Coizet V, Pernet-Gallay K (2019). Implication of anterior nucleus of the thalamus in mesial temporal lobe seizures. Neuroscience.

[15] Romeo A, Issa Roach AT, Toth E (2019). Early ictal recruitment of midline thalamus in mesial temporal lobe epilepsy. Ann. Clin. Transl. Neurol..

[16] Pergola G, Ranft A, Mathias K (2013). The role of the thalamic nuclei in recognition memory accompanied by recall during encoding and retrieval: An fMRI study. NeuroImage.

[17] Morgan VL, Rogers BP, Abou-Khalil B (2015). Segmentation of the thalamus based on BOLD frequencies affected in temporal lobe epilepsy. Epilepsia.

[18] Yakushev I, Drzezga A, Habeck C (2017). Metabolic connectivity: methods and applications. Curr. Opin. Neurol..

[19] Sala A, Lizarraga A, Ripp I (2022). Static versus functional PET: Making sense of metabolic connectivity. Cereb Cortex.

[20] Jha A, Teotonio R, Smith AL (2020). Metabolic lesion-deficit mapping of human cognition. Brain.

[21] Dabbs K, Jones J, Seidenberg M (2009). Neuroanatomical correlates of cognitive phenotypes in temporal lobe epilepsy. Epilepsy Behav..

[22] Aldenkamp A, Arends J (2004). The relative influence of epileptic EEG discharges, short nonconvulsive seizures, and type of epilepsy on cognitive function. Epilepsia.

[23] Park HJ, Friston K (2013). Structural and functional brain networks: From connections to cognition. Science.

[24] Pulvermuller F, Tomasello R, Henningsen-Schomers MR (2021). Biological constraints on neural network models of cognitive function. Nat. Rev. Neurosci..

[25] Le TM, Huang AS, O'Rawe J (2020). Functional neural network configuration in late childhood varies by age and cognitive state. Dev. Cogn. Neurosci..

[26] Allone C, Lo Buono V, Corallo F (2017). Neuroimaging and cognitive functions in temporal lobe epilepsy: A review of the literature. J. Neurol. Sci..

[27] Hermann B, Conant LL, Cook CJ (2020). Network, clinical and sociodemographic features of cognitive phenotypes in temporal lobe epilepsy. NeuroImage Clin..

[28] Laurent A, Artiges E, Mellerio C (2020). Metabolic correlates of cognitive impairment in mesial temporal lobe epilepsy. Epilepsy Behav..

[29] Caciagli L, Allen LA, He X, Trimmel K, Vos SB, Centeno M (2020). Thalamus and focal to bilateral seizures: A multiscale cognitive imaging study. Neurology.

[30] Mueller SG, Laxer KD, Cashdollar N (2006). Voxel-based optimized morphometry (VBM) of gray and white matter in temporal lobe epilepsy (TLE) with and without mesial temporal sclerosis. Epilepsia.

[31] Marcian V, Marecek R, Koritakova E (2018). Morphological changes of cerebellar substructures in temporal lobe epilepsy: A complex phenomenon, not mere atrophy. Seizure.

[32] Park KM, Kim TH, Mun CW (2018). Reduction of ipsilateral thalamic volume in temporal lobe epilepsy with hippocampal sclerosis. J. Clin. Neurosci..

[33] Jber M, Habibabadi JM, Sharifpour R (2021). Temporal and extratemporal atrophic manifestation of temporal lobe epilepsy using voxel-based morphometry and corticometry: Clinical application in lateralization of epileptogenic zone. Neurol. Sci..

[34] Barron DS, Tandon N, Lancaster JL (2014). Thalamic structural connectivity in medial temporal lobe epilepsy. Epilepsia.

[35] Rosenberg DS, Mauguiere F, Demarquay G (2006). Involvement of medial pulvinar thalamic nucleus in human temporal lobe seizures. Epilepsia.

[36] Bertram EH (2014). Extratemporal lobe circuits in temporal lobe epilepsy. Epilepsy Behav..

[37] Taylor J, Kolamunnage-Dona R, Marson AG (2010). Patients with epilepsy: Cognitively compromised before the start of antiepileptic drug treatment?. Epilepsia.

[38] McMillan TM, Mason CA, Seidenberg M (2021). The impact of processing speed on cognition in temporal lobe epilepsy. Epilepsy Behav..

[39] Dow C, Seidenberg M, Hermann B (2004). Relationship between information processing speed in temporal lobe epilepsy and white matter volume. Epilepsy Behav..

[40] Hwang G, Dabbs K, Conant L (2019). Cognitive slowing and its underlying neurobiology in temporal lobe epilepsy. Cortex.

[41] Fama R, Sullivan EV (2015). Thalamic structures and associated cognitive functions: Relations with age and aging. Neurosci. Biobehav. Rev..

[42] Steiner L, Federspiel A, Slavova N (2020). Functional topography of the thalamo-cortical system during development and its relation to cognition. NeuroImage.

[43] Clark BJ, Harvey RE (2016). Do the anterior and lateral thalamic nuclei make distinct contributions to spatial representation and memory?. Neurobiol. Learn. Mem..

[44] Li R, Zhang L, Guo D (2021). Temporal lobe epilepsy shows distinct functional connectivity patterns in different thalamic nuclei. Brain Connect..

[45] Hughes EJ, Bond J, Svrckova P (2012). Regional changes in thalamic shape and volume with increasing age. NeuroImage.

[46] Bernhardt BC, Bernasconi N, Kim H (2012). Mapping thalamocortical network pathology in temporal lobe epilepsy. Neurology.

[47] Benedek K, Juhasz C, Muzik O (2004). Metabolic changes of subcortical structures in intractable focal epilepsy. Epilepsia.

[48] Delorenzo RJ, Sun DA, Deshpande LS (2005). Cellular mechanisms underlying acquired epilepsy: the calcium hypothesis of the induction and maintainance of epilepsy. Pharmacol. Ther..

[49] Blazquez E, Hurtado-Carneiro V, LeBaut-Ayuso Y (2022). Significance of brain glucose hypometabolism, altered insulin signal transduction, and insulin resistance in several neurological diseases. Front Endocrinol.

[50] Akimura T, Yeh HS, Mantil JC (1999). Cerebral metabolism of the remote area after epilepsy surgery. Neurol. Med. Chir..

[51] Zhao Y, Zhang C, Yang H (2021). Recovery of cortical atrophy in patients with temporal lobe epilepsy after successful anterior temporal lobectomy. Epilepsy Behav..

[52] Li W, Jiang Y, Qin Y (2022). Cortical remodeling before and after successful temporal lobe epilepsy surgery. Acta Neurol. Scand..

[53] Quon RJ, Mazanec MT, Schmidt SS (2020). Antiepileptic drug effects on subjective and objective cognition. Epilepsy Behav..

[54] Cain SM, Snutch TP (2013). T-type calcium channels in burst-firing, network synchrony, and epilepsy. Biochim. Biophys. Acta.

