# Supplementary figures and images for: Morphological and metabolic asymmetries of the thalamic subregions in temporal lobe epilepsy predict cognitive functions
Source: Sci Rep. 2023 Dec 18;13:22611. doi: 10.1038/s41598-023-49856-x (PMC10730825; doi:10.1038/s41598-023-49856-x)

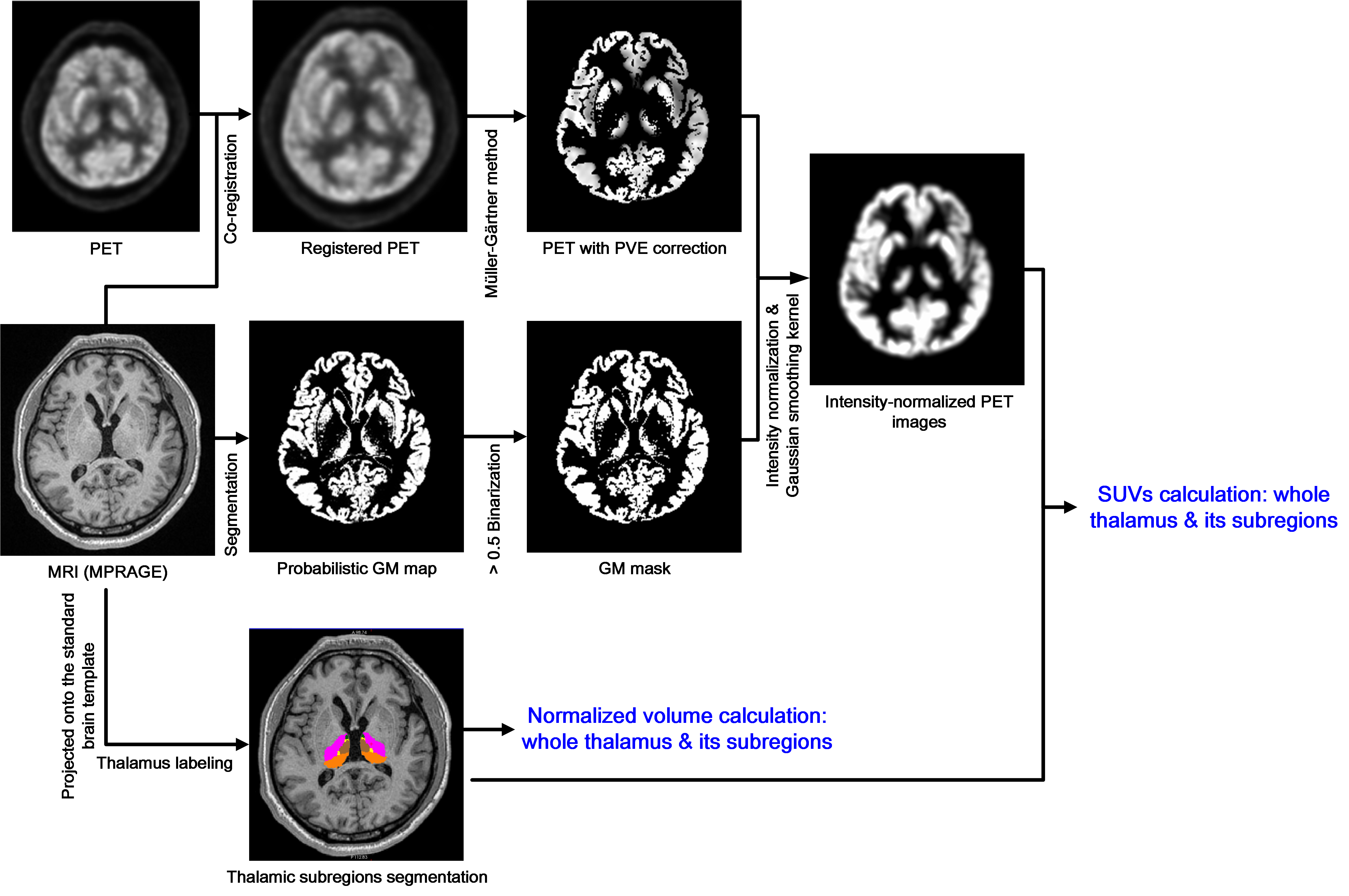

Supplement: Supplementary file 1 — Supplementary Figure 1. [file 41598_2023_49856_MOESM1_ESM.tiff]
