# Supplementary material for: Morphological and metabolic asymmetries of the thalamic subregions in temporal lobe epilepsy predict cognitive functions
Source: Sci Rep. 2023 Dec 18;13:22611. doi: 10.1038/s41598-023-49856-x (PMC10730825; doi:10.1038/s41598-023-49856-x)
Supplement: Supplementary file 2 — Supplementary Information 2. [file 41598_2023_49856_MOESM2_ESM.docx]

**Image processing and Thalamus segmentation**

The ^18^F-FDG PET images were co-registered with the MPRAGE images, according to the 3-D voxel-based, normalized mutual information method, using Statistical Parametric Mapping 12 (SPM12). The Müller-Gärtner method was used to correct for the partial volume effect of the registered ^18^F-FDG PET images in the PETPVE12 Toolbox.^1^ The MPRAGE images were used for segmentation, dividing into the probabilistic gray matter (GM) map using SPM12. The entire brain GM was defined when the intensity probability exceeding 0.5, and then binarized, resulting in the GM mask. The SUVs of GMs from the entire brain were used as a reference value for intensity normalization to quantify the registered ^18^F-FDG PET images. The normalized ^18^F-FDG PET images were spatially smoothed using a Gaussian smoothing kernel with 6-mm full width at half maximum.

The MPRAGE images were projected onto the standard brain template, and then the thalamus was automatically partitioned using FreeSurfer (version 7.1.1). The standard FreeSurfer “recon-all” processing stream was used to provide the morphometry data of the thalamic images for each patient. We had used the officially provided code from the website to correct this possible false of original version. (https://freesurfer.net/fswiki/ThalamicNuclei).The entire individual thalamus was first partitioned from the MPRAGE images by automatic labeling, and then the adaptive segmentation technique was applied for further segmentation into the six thalamic subnuclei and volume calculation for quantitative analysis.^2^ **Figure 1** shows the anatomical locations of each thalamic subfield in MPRAGE images in a patient with TLE. There were anterior (anteroventral), lateral (laterodorsal and lateral posterior), ventral (ventral anterior, ventral anterior magnocellular, ventral lateral anterior, ventral lateral posterior, ventral posterolateral, and ventromedial), intralaminar (central medial, central lateral, paracentral, centromedian, and parafascicular), medial (paratenial, reuniens (medial ventral), mediodorsal medial magnocellular, and mediodorsal lateral parvocellular), and posterior (lateral geniculate, medial geniculate, limitans (suprageniculate), pulvinar anterior, pulvinar medial, pulvinar lateral, and pulvinar inferior). The SUVs of the six subregions were extracted from the fused GM map, and then normalized by divided by the average SUV values of whole GM (SUV ratio, SUVR). Thus, the values of both the volume and the SUVR of the entire thalamus and individual thalamic subnuclei were computed.

**Supplementary Figure 1. Flowchart of the processing steps of the MRI and PET images.**

First row: The PET images were coregistered with the MPRAGE images using Statistical Parametric Mapping 12 (SPM12). Then, the Müller-Gärtner method was used to correct for the partial volume effect of the registered PET-MRI images. Second row: The MPRAGE images were used to generate the probabilistic gray matter (GM) mask using SPM12. The SUVs of GMs from the entire brain were used as a reference value for intensity normalization of the registered PET images, generating standard uptake value ratio (SUVR). Then, the registered PET images of the GM were spatially smoothed using a Gaussian smoothing kernel. Third row: The MPRAGE images were projected onto the standard brain template, and then the thalamus was automatically segmented using FreeSurfer. It was further segmented into the six thalamic subnuclei for volume and SUVR quantitation analysis

**Reference:**

1. Muller-Gartner HW, Links JM, Prince JL, et al **Measurement of radiotracer concentration in brain gray matter using positron emission tomography: MRI-based correction for partial volume effects.** *J Cereb Blood Flow Metab* 1992:12:571-83.
2. Iglesias JE, Insausti R, Lerma-Usabiaga G, *et al*  **A probabilistic atlas of the human thalamic nuclei combining ex vivo MRI and histology.** *NeuroImage* 2018:183:314-26.
